# Green tide development associated with submarine groundwater discharge in a coastal harbor, Jeju, Korea

**DOI:** 10.1038/s41598-017-06711-0

**Published:** 2017-07-24

**Authors:** Hyeong Kyu Kwon, Hyekyung Kang, Yong Hwa Oh, Sang Rul Park, Guebuem Kim

**Affiliations:** 1 School of Earth and Environmental Sciences, Seoul National University, Seoul, 08826 Republic of Korea; 2 Department of Marine Life Sciences, Jeju National University, Jeju, 63243 Republic of Korea

## Abstract

We measured the magnitude of submarine fresh groundwater discharge (SFGD) and associated nutrient inputs to Jocheon harbor, on Jeju Island, Korea, during four sampling periods, in order to determine the link between SFGD and *Ulva* sp. green tide development. Good correlations among salinity, ^222^Rn, and dissolved inorganic nitrogen (DIN) in harbor seawater suggest that SFGD is the major source of DIN and fresh water since there are no surface runoffs. Using a ^222^Rn mass balance model, SFGD to the harbor was estimated to be 5.8 ± 2.3 × 10^4^ m^3^ d^−1^. The DIN inputs through SFGD enhanced DIN concentrations in harbor seawater approximately 10-fold of those in the open-ocean (outer harbor) seawater. Results from mesocosm experiments showed that the growth rate of *U. pertusa* increased by 160% on average due to the enhanced DIN concentrations (from 1 to 24 µM) through SFGD in this harbor. Thus, we conclude that DIN inputs through SFGD cause the green tide development in Jocheon harbor and perhaps in other green tide regions where river inputs are absent.

## Introduction

In recent years, there has been an increasing number of reports on the occurrence of massive green tides worldwide^1–5^. In general, the genus *Ulva* has been reported as the dominant species leading to green tides^6, 7^ due to the morphological characteristics of its thalli, rapid uptake of nutrients, and fast growth rates^8, 9^. These blooms of opportunistic macroalgae are generally explained by eutrophication caused by anthropogenic nutrient loads^2–5, 10^. Liu *et al*.^2^ suggested that the seawater contamination by wastewater, agricultural, and aquacultural discharges could cause green tides in the western Yellow Sea. As such, Nelson *et al*.^10^ suggested that anthropogenic inputs of nutrients from urbanized areas through wastewater led to the outbreak of green tides in the Salish Sea, Canada. Nutrient inputs from rivers also resulted in the development of green tides in the Saint-Brieuc Bay (France), Newport Bay (USA), and Palmones estuary (Spain)^3–5^.

In the eastern coast of Jeju Island, green tides of *Ulva* spp. (*U. conglobata* and *U. pertusa*) have occurred annually since the early 2000s^11^. The amount of green macroalgal biomass produced annually is reported to be up to 7 kg m^−2^ (wet weight)^11^. These green tides have resulted in aesthetic problems and noxious smells deposits of drift green macroalgae on shorelines. The occurrence of the green tides in the eastern coast of Jeju has been speculated to be due to marine environmental changes (e.g., pollution and global warming) together with geological factors. The eastern coast of Jeju Island is shallow, semi-enclosed, and sandy, whereas the western coast is hilly, rocky, and deep. Since there are no distinctive rivers in this island, submarine groundwater discharge (SGD) is thought to be the major source of nutrients throughout the entire coast of Jeju^12^.

SGD includes submarine fresh groundwater discharge (SFGD) and submarine saline groundwater (i.e., recirculating seawater) discharge. Since there is no tool to directly gauge SGD over a large area, ^222^Rn has been used as one of the most powerful SGD tracers in coastal waters^12–15^. It is about two to four orders of magnitude more concentrated in groundwater than in surface waters, conservative, and known to decay at a rate (t_1/2_ = 3.82 days) comparable to the time scale of coastal waters^16–18^. In general, SGD has been considered as an important source of natural radionuclides, carbon, metals, and nutrients to the coastal areas^13, 19–21^. Excess nutrient inputs through SGD can result in eutrophication in coastal areas, which can cause harmful algal blooms^22–24^. It has been reported that SGD can influence biological communities by regulating salinity, temperature, nutrient levels, N:P ratio, CO_2_, and dissolved organic substances^22, 24, 25^.

**Figure 1:**
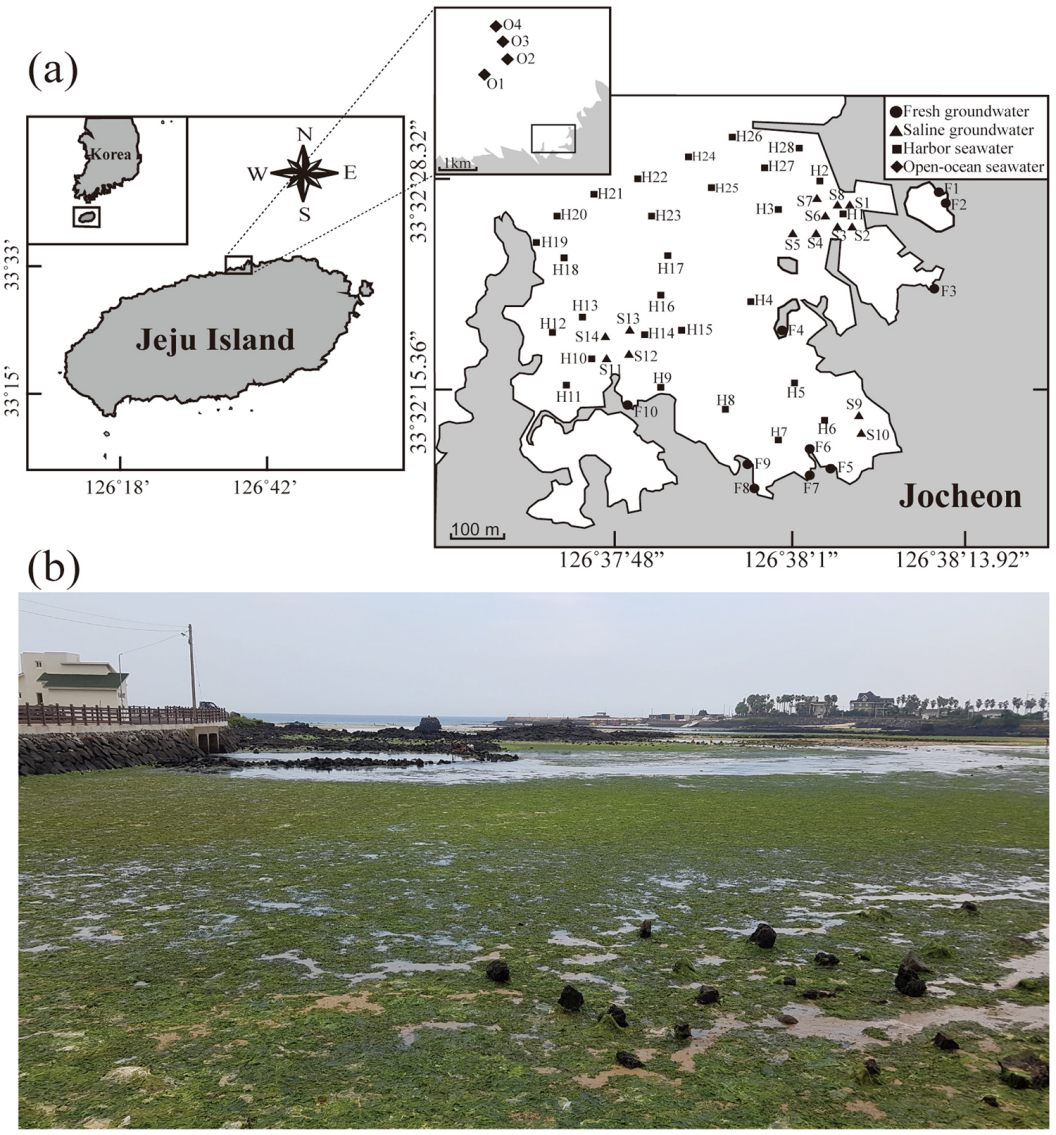
(**a**) The maps showing the sampling stations in coastal area of Jocheon, Jeju Island. The filled circles, triangles, squares and diamonds represent fresh groundwater (Sts. F1-F10), saline groundwater (Sts. S1-S14), harbor seawater (Sts. H1-H28), and open-ocean seawater (Sts. O1-O4), respectively. Maps were created using Adobe Illustrator CC (http://www.adobe.com/illustrator). (**b**) Photograph of massive green tide development in Jocheon, Jeju Island.

In Bangdu Bay (located in the southeastern part of Jeju), where saline groundwater discharge is pronounced, excess nutrient inputs through SGD have resulted in benthic eutrophication^10^. Hwang *et al*.^12^ suggested a possible connection between the high nutrient inputs from saline groundwater and benthic eutrophication in Bangdu Bay. However, this hypothesis has not yet been tested in SFGD dominated areas. Thus, in this study, we determine the link between SFGD and *Ulva* sp. green tides occurring in Jocheon harbor in the northeastern part of Jeju Island, where SFGD dominates (Fig. 1). In order to achieve our goals, we assessed (1) the magnitude of SFGD, (2) SFGD-driven nutrient fluxes to the harbor, and (3) effects of SFGD on the growth of *U. pertusa* through mesocosm experiments. According to laboratory culture experiment results by Choi^26^, the growth rate of *U. pertusa* is dependent on dissolved inorganic nitrogen (DIN) but is not varied for dissolved inorganic phosphorus (DIP) concentrations between 0.03 and 22 µM. Thus, based on these results, we conducted mesocosm experiments for different proportions of fresh groundwater in order to look at the link between SFGD and *Ulva* sp. green tides.

## Results and Discussion

### Distributions of salinity, ^222^Rn, and nutrients in groundwater and seawater

**Figure 2:**
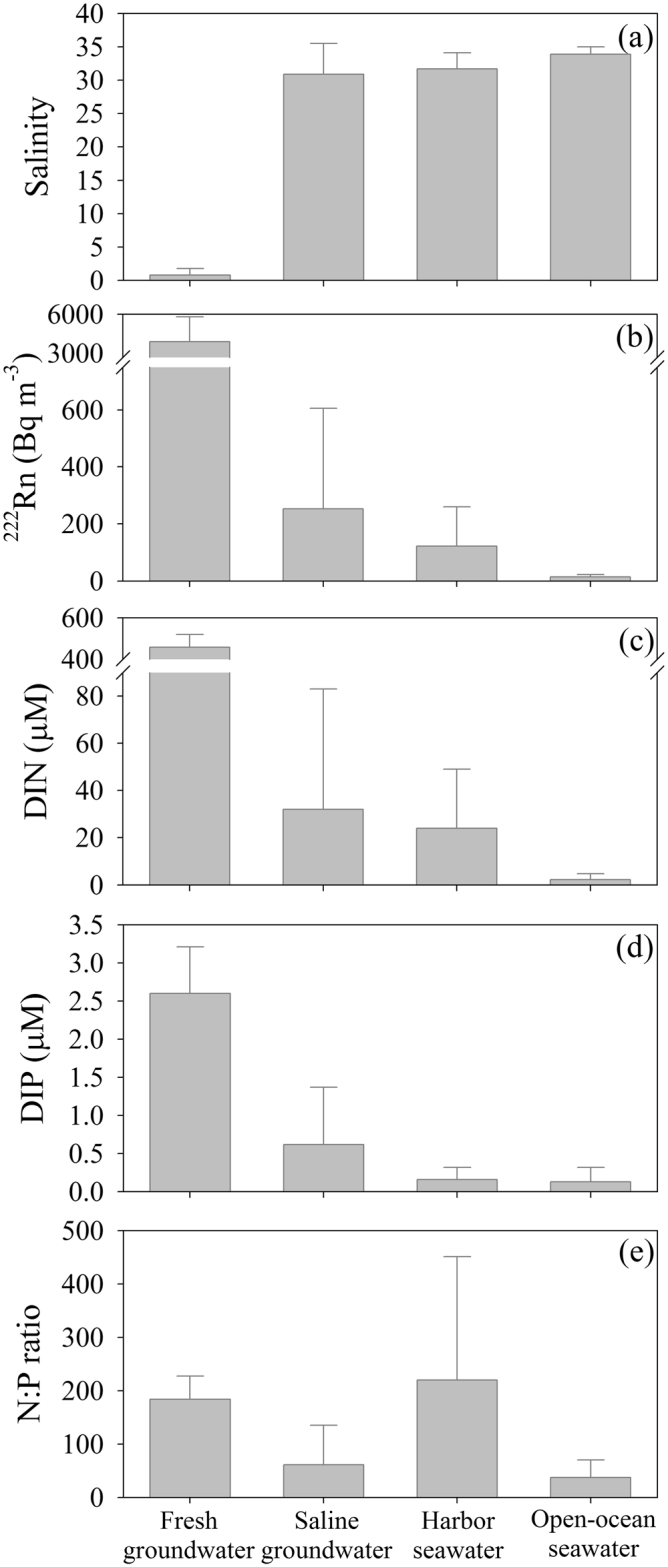
The averages and standard deviations of (**a**) salinity, (**b**) ^222^Rn, (**c**) DIN, (**d**) DIP concentrations, and (**e**) N:P ratios in fresh groundwater, saline groundwater, harbor seawater, and open-ocean seawater from July 2015 to March 2016 in Jocheon, Jeju Island. The error bars are the standard deviation of each data set.

The salinities in spring water (fresh groundwater), seeping groundwater (saline groundwater), harbor seawater, and open-ocean seawater were in the range of 0.2–4.6 (average: 0.8 ± 1.0), 15.0–34.4 (average: 30.9 ± 4.6), 23.8–34.7 (average: 31.7 ± 2.4), and 32.9–34.8 (average: 33.9 ± 1.1), respectively (Fig. 2a). There were no significant seasonal differences in salinities for fresh groundwater, saline groundwater, and open-ocean seawater (ANOVA, F(9,44) = 198, *p* > 0.05). The salinities in harbor seawater were significantly lower in summer (July and August) than those in the other sampling periods (ANOVA, F(3,76) = 14, *p* < 0.05, Supplementary Tables S1–S4). Since groundwater residence time is shorter than five years in the entire Jeju Island^27^, this lower salinity might be associated with larger groundwater inputs as well as direct rainfall to the sea following heavy summer precipitations (>60% of the precipitation occurs in the summer monsoon season^28^). The salinities in bottom seawater were significantly higher than those in surface seawater and slightly higher than those in saline groundwater (ANOVA, F(2,98) = 9.9, *p* < 0.05, Supplementary Tables S1–S4). The saline groundwater in the seepage chambers seems to be composed of approximately 10% fresh groundwater and 90% recirculated seawater.

The activities of ^222^Rn in fresh groundwater, saline groundwater, harbor seawater, and open-ocean seawater were in the range of 1400–8270 (average: 3900 ± 1900 Bq m^−3^), 30–1070 (average: 250 ± 35 Bq m^−3^), 5–740 (average: 120 ± 140 Bq m^−3^), and 4–24 Bq m^−3^ (average: 14 ± 8 Bq m^−3^), respectively (Fig. 2b). The activities of ^222^Rn in surface seawaters of harbor were significantly higher than those in bottom seawaters (ANOVA, F(1,75) = 11, *p* < 0.05, Supplementary Tables S1–S4). This result indicates that the fresh groundwater, which originally had a very high level of ^222^Rn activities, was horizontally mixed into surface seawaters. The activities of ^222^Rn in saline groundwater were an order of magnitude lower than those in fresh groundwater, perhaps due to rapid recirculation of seawater through porous rocks and sediments.

The DIN concentrations in fresh groundwater were in the range of 380–650 µM (average: 460 ± 60 µM), which were two orders of magnitude higher than those in open-ocean seawater during the four sampling periods (Fig. 2c). As such, the DIN concentrations in harbor seawater were an order of magnitude higher than those in open-ocean seawater, indicating that there are significant DIN inputs into the harbor. The significant correlation between DIN concentrations in water samples (fresh groundwater and seawater) and salinity (Supplementary Figure S1) suggests that the major source of DIN in the harbor is fresh groundwater. It is notable that the concentrations of DIN presented no significant differences between saline groundwater and harbor seawater (ANOVA, F(1,99) = 0.99, *p* > 0.05), indicating that there was no addition of DIN by saline groundwater.

The DIP concentrations in fresh groundwater were in the range of 1.9–4.3 µM (average: 2.6 ± 0.6 µM), which were an order of magnitude higher than those in open-ocean seawater during the four sampling periods (Fig. 2d). However, the DIP concentrations were similar for the harbor and open-ocean seawaters, indicating that the input of DIP in the harbor was insignificant. The correlation between DIP and salinity for water samples (fresh groundwater and seawater) showed large scattering (Supplementary Figure S1). The DIP concentrations in some stations were higher than the mixing line between fresh groundwater and open-ocean seawater, while those in the other stations were lower than the mixing line. This result suggests that there are additional sources of DIP in this harbor (i.e., input of regenerated DIP through saline groundwater) and the removal by biological uptake.

The N:P ratios in fresh groundwater, saline groundwater, harbor seawater, and open-ocean seawater were in the range of 108–260 (average: 184 ± 44), 12–262 (average: 62 ± 77), 7–1013 (average: 217 ± 229) and 12–60 (average: 30 ± 23), respectively (Fig. 2e). Our results suggest that higher N:P ratios, relative to the Redfield ratio (16), in harbor seawater is associated with larger inputs of N relative to P via SFGD. Although these higher ratios may limit the growth of phytoplankton in seawater, they could be more favorable for the growth of *U. pertusa* ^26^.

### Determination of SFGD

Since SFGD is found to be the major source of nutrients in this harbor, we determine the magnitude of SFGD using a ^222^Rn tracer. The activities of ^222^Rn in harbor samples showed a significant correlation with salinities (Supplementary Figure 1S), indicating that the main source of ^222^Rn, together with nutrients, is SFGD. Thus, we estimated the SFGD using a ^222^Rn mass balance model in this region.

Since there is no river discharge in the harbor, under steady-state conditions, the ^222^Rn mass balance model can be expressed as follows (Eq. 1)^13^:

1 $${{\rm{F}}}_{{\rm{Diff}}}+{{\rm{C}}}_{{\rm{FGW}}}\times {{\rm{A}}}_{{\rm{Bott}}}\times {{\rm{\Psi }}}_{{\rm{SFGD}}}-{{\rm{I}}}_{{\rm{SW}}}\times {{\rm{\lambda }}}_{\mathrm{Rn} \mbox{-} 222}-{{\rm{C}}}_{{\rm{EX}}}\times {{\rm{V}}}_{{\rm{S}}}\times {{\rm{\lambda }}}_{{\rm{Mix}}}-{{\rm{F}}}_{{\rm{Atm}}}=0$$

where F_Diff_ is the diffusive fluxes from bottom sediments and C_FGW_ is the concentrations of ^222^Rn in fresh groundwater. Here, C_FGW_ is calculated by extrapolating harbor seawater concentrations. The extrapolated concentrations of ^222^Rn appear to be reasonable since spring water samples showed large scattering (Supplementary Figure S1), and the relative contributions from different springs are unknown. A_Bott_ is the bottom area of the harbor, Ψ_SFGD_ is the seepage rate of fresh groundwater, I_SW_ is the ^222^Rn inventory in harbor seawater, λ_Rn-222_ is the decay constant of ^222^Rn, C_EX_ is the difference in the ^222^Rn concentrations between harbor seawater and open-ocean seawater, V_S_ is the volume of harbor seawater, λ_Mix_ is the mixing rate of harbor seawater with the open-ocean seawater (reciprocal of the water residence time), and F_Atm_ is the evasive fluxes to the atmosphere. The second term is the fluxes from SFGD, the third term is the output fluxes by radioactive decay, and the forth term is the output fluxes by mixing with the open-ocean seawater. The values for each term used in the ^222^Rn mass balance model are shown in Table 1.

**Table 1:** The terms and values used in the simultaneous equation for the ^222^Rn mass balance in Jocheon harbor, Jeju Island during July 2015, August 2015, February 2016, and March 2016.

Term (unit)	July 2015	Aug. 2015	Feb. 2016	Mar. 2016
A_Bott_ (m^2^)	3.2 × 10^5^			
V_S_ (m^3^)	7.4 × 10^5^			
F_Diff_ (Bq d^−1^)	5.3 × 10^5^			
C_FGW_ (Bq m^−3^)	1910			
F_Decay_ (I_SW_ × λ_Rn-222_) (Bq d^−1^)	2.5 × 10^7^	1.4 × 10^7^	8.8 × 10^6^	1.9 × 10^7^
C_EX_ (Bq m^−3^)	170	96	50	128
λ_mix_ (d^−1^)	1.00	1.19	1.56	0.78
F_Atm_ (Bq d^−1^)	2.3 × 10^7^	6.8 × 10^6^	3.6 × 10^6^	7.4 × 10^6^

The residence times of seawater (T_W_ = 1/λ_Mix_) were estimated using the tidal prism method^29, 30^. The result from this method is a function of the tidal range and the volume of harbor seawater. Assuming that return flow is negligible since the offshore northeastward current velocity is high (10–15 cm s^−1^) along the coast of Jeju Island^31^, the residence times in the harbor were estimated to be 1.0 day, 0.8 days, 0.6 days and 1.3 days in July 2015, August 2015, February 2016, and March 2016, respectively.

On the basis of the ^222^Rn mass balance model, SFGD were estimated to be 9.1 × 10^4^, 5.4 × 10^4^, 3.6 × 10^4^, and 5.2 × 10^4^ m^3^ d^−1^ in July 2015, August 2015, February 2016, and March 2016, respectively. The calculation methods used and the assumptions made for calculating the values of the mixing rates and diffusion from bottom sediments were the same as those in Hwang *et al*.^12^. In this study, the atmospheric loss of ^222^Rn (F_Atm_) was calculated from the equation proposed by MacIntyre *et al*.^32^.

### SFGD-driven nutrient fluxes

**Figure 3:**
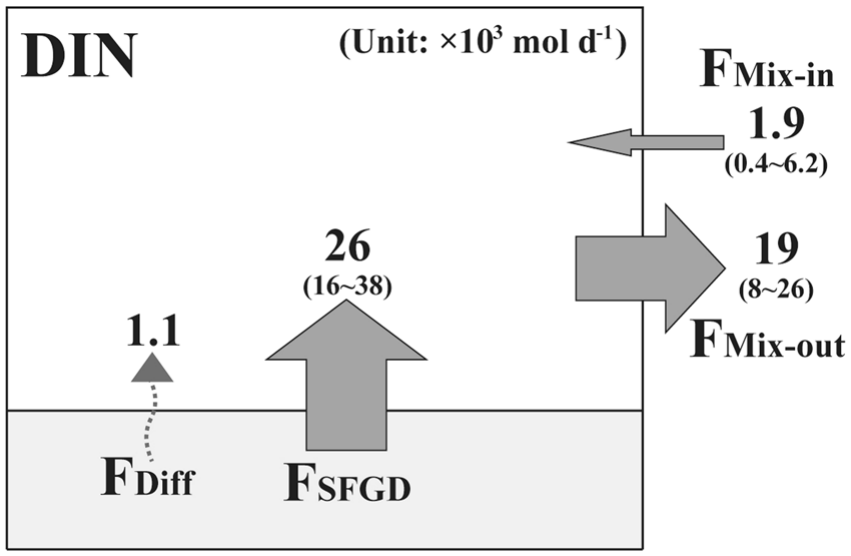
The DIN budget showing the DIN fluxes (×10^3^ mol d^−1^) though fresh groundwater discharge, diffusion from bottom sediments, and mixing between harbor seawater and open-ocean seawater from July 2015 to March 2016 in Jocheon harbor, Jeju Island.

The DIN flux through SFGD (F_SFGD_) was determined by multiplying the SFGD during each sampling period by the average concentration of DIN in fresh groundwater. The average concentrations of DIN (447 µM) and DIP (2.4 µM) in all groundwater samples are similar to those in the groundwater samples corresponding to the major springs mentioned earlier based on the ^222^Rn extrapolation from harbor seawaters (Supplementary Figure S1). Since nutrients are not conservative in seawater, we used the average concentrations in all groundwater for the SFGD-driven flux estimations. Then, the DIN fluxes through SFGD were calculated to be 38 × 10^3^, 28 × 10^3^, 16 × 10^3^, and 23 × 10^3^ mol d^−1^ in July 2015, August 2015, February 2016, and March 2016, respectively (Fig. 3). The DIN flux through SFGD was higher in summer relative to those in other seasons due to the higher magnitude of SFGD.

The DIN flux through the diffusion from bottom sediments (F_Diff_) was determined by multiplying the total area of the harbor by the regeneration rate of DIN using globally available data on sandy sediments (Supplementary refs. 1–9). The regeneration rate of DIN ranged from 4 to 3410 µmol m^−2^ d^−1^ (average: 879 ± 962 µmol m^−2^ d^−1^). If we apply the maximum diffusive flux, the flux of DIN through the diffusion from bottom sediments was calculated to be 1.1 × 10^3^ mol d^−1^ (Fig. 3), which was lower than 5% of the total DIN flux.

The DIN flux to the open-ocean (F_Mix_) was determined by dividing inventory of excess DIN by the water residence time of harbor seawater. Here, the inventory of excess DIN was determined by multiplying the volume of harbor seawater with the difference in the averages of DIN concentrations between harbor seawater and open-ocean seawater. The average flux of DIN through the input of open-ocean seawater (F_Mix-in_) during the study periods was estimated to be 2.0(±2.8) × 10^3^ mol d^−1^ (Fig. 3). Although the contribution of F_Mix-in_ to the total DIN flux was relatively higher in February 2016 (32%), that was less than 10% in the other periods. The average flux of DIN to the outer harbor (F_Mix-out_) during the study periods was estimated to be 19(±8) × 10^3^ mol d^−1^ (Fig. 3). These results indicate that the flux of DIN through SFGD enhanced approximately 10-fold of DIN level in harbor seawater. Based on these budget calculations, the flux of DIN through SFGD contributes approximately 71–98% (average: 90 ± 13%) of the total DIN flux. The concentrations of DIN in aquacultural discharging water in the eastern coast of Jeju were <50 µM^33^, which were an order of magnitude lower than those in fresh groundwater. In addition, the significant correlations between DIN and salinity in harbor seawater (Supplementary Figure S1), together with DIN mass balances, confirms that there are no additional local sources, except SFGD. Although there may be large uncertainties in this estimate, it is obvious that SFGD is a major source of DIN in the harbor seawater.

The DIP flux through SFGD was determined by multiplying the SFGD during each sampling period by the average concentration of DIP in fresh groundwater. The DIP fluxes through SFGD were calculated to be 0.21 × 10^3^, 0.15 × 10^3^, 0.09 × 10^3^, and 0.14 × 10^3^ mol d^−1^ in July 2015, August 2015, February 2016, and March 2016, respectively. The DIP flux through SFGD was higher in summer relative to those in the other seasons due to the higher magnitude of SFGD. However, as shown earlier, the inputs of DIP via SFGD are insignificant relative to DIN, and there are significant DIP inputs from unknown sources (i.e., saline groundwater discharge). Moreover, the difference in DIP concentrations between the harbor and open-ocean seawater is unclear, perhaps due to biological removal inside the harbor. Therefore, in this study, we do not establish a mass balance model for DIP.

Based on the mass balance of DIN, the input flux of DIN to the harbor seawater was approximately 50% larger than the output flux (Fig. 3), perhaps due to removal by biological assimilation. If the C:N ratio of 16:1 is applied for the average uptake rate of *U. pertusa* ^26^ for the removed DIN, the total production of carbon is calculated to be 1.9 × 10^6^ gC d^−1^ in harbor seawater. This amount corresponds to 790 t wet weight (2.5 kg m^−2^) of *Ulva* sp. biomass if the average carbon uptake rate of *U. pertusa* (0.2 mmol g^−1^ d^−1^) reported by Choi^26^ is applied. This production is within the range of <7 kg m^−2^ of *Ulva* sp. production in this region^11^. Thus, DIN input through SFGD seems to be balanced by the total production of *Ulva* sp. in this harbor.

### Effects of SFGD on the growth of *U. pertusa*

Spatial patterns of green tides can be affected by various factors, including temperature, salinity, irradiance, nutrient conditions, competition, grazer preferences, geomorphology, and current velocity^8, 26, 34, 35^. Amongst these factors, temperature and irradiance are the most important factors in determining uptake of nutrients and growth rates^34^. Choi^26^ reported that *U. pertusa* adapted to a broad range of temperature and irradiance conditions. The viable temperature range was between 10 and 25 °C, with the growth optimal temperature being 20 °C. In the case of irradiance, growth of *U. pertusa* saturated between 100 and 320 µmol m^−2^ s^−1^. During our mesocosm experiments, the temperature and irradiance ranged from 16.2 to 18.3 °C and from 153 to 1471 µmol m^−2^ s^−1^, respectively (Table 2).

**Table 2:** The initial conditions of temperature, irradiance, salinity, and nutrients at different mixing ratios between open-ocean seawater and fresh groundwater (FGW) from April 26 to April 30, 2016, in mesocosm experiments.

	Open-ocean seawater	FGW 1%	FGW 5%	FGW 10%	FGW 30%	FGW 50%
Temperature (°C)	16.2–18.3					
Irradiance (µmol m^−2^ s^−1^)^*^	153–1471					
Salinity	34.6	33.8	32.3	30.4	24.1	17.3
DIN (µM)	1.0	6.2	21	40	116	184
DIP (µM)	0.07	0.10	0.15	0.36	0.64	1.19

*Irradiance data were obtained from Korea Meteorological Administration.

**Figure 4:**
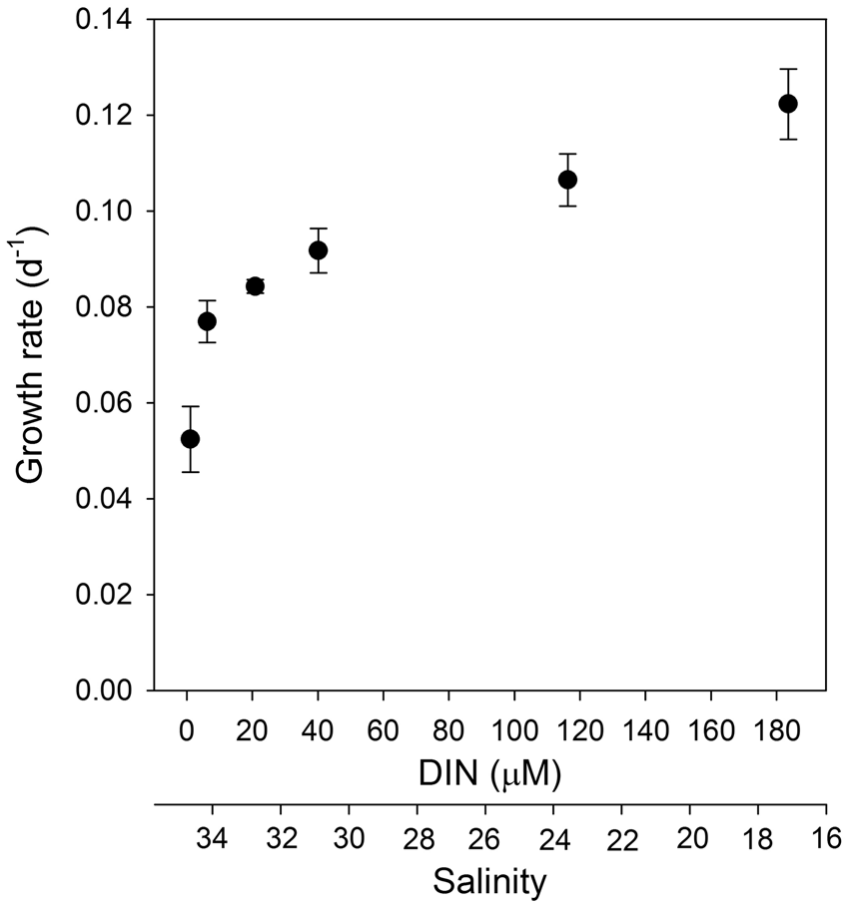
The growth rate of *U. pertusa* as a function of DIN concentrations and salinities by adding different amounts of fresh groundwater.

The growth rates of *U. pertusa* from our mesocosm experiments were plotted against the variations of DIN and salinity by adding different amounts of fresh groundwater (Fig. 4). The growth rates increased by up to 240% for the range of fresh groundwater addition in this experiment, with an average growth rate of 160% for the average DIN of harbor seawater observed. According to laboratory culture experiment results by Choi^26^, the growth rates of *U. pertusa* occurring in this region increased approximately 290% for DIN concentrations from 2 to 191 µM, although no variations were observed for DIP concentrations between 0.03 and 22 µM. The highest growth rates were observed at a nutrient level of 30 µM for DIN and 0.8 µM for DIP. As such, the tissue N:P ratio was about 60 at the highest growth rate, indicating that N is more important for the growth of this species. In addition, unusually lower (<10) or higher (>35) salinity conditions negatively affect the nutrient uptake and growth of *Ulva* sp.^26, 36^. Choi^26^ reported that under no limitation conditions for nutrients and irradiance, *U. pertusa* showed higher growth rates up to 170% within the salinity between 15 and 25, with an optimum growth rate in the range of 15 to 20, for wide salinity range (5–40) condition experiments.

Our mesocosm experiment results, together with the laboratory experiment results by Choi^26^, suggest that the sharp increase of growth rate up to 0.09 d^−1^ (at a DIN level of 30 µM and salinity of 31) is predominantly due to DIN addition by fresh groundwater (Fig. 4). For further fresh groundwater additions (salinity <31), both DIN and salinity seem to enhance the growth rate. The salinities in the innermost areas of the harbor were lower down to 23.8 (Supplementary Table S1). Thus, both salinity and DIN seem to play an important role on forming green tides in the inner part of the harbor. In addition, higher enrichment of DIN, relative to DIP, in harbor seawater due to the discharge of fresh groundwater (with N:P ratios between 108 and 256), together with a low current velocity (<10 cm s^−1^) in the inner harbor^37, 38^, seems to provide a more favorable environment for the growth of *U. pertusa*.

## Conclusions

Inputs of nutrients via SFGD enhanced the level of nutrients in harbor seawater by an order of magnitude higher. Based on mesocosm experiments, the growth rate of *U. pertusa* increased by up to 240% due to excess DIN inputs and decreased salinities in association with large SFGD. Thus, excess DIN inputs and salinity decreases through SFGD seem to result in the development of green tides in Jocheon harbor. Further studies are necessary to determine the main source of anthropogenic nutrients in groundwater and modeling the development of green tides in association with SFGD and nutrient delivery, together with other environmental factors, particularly at germling and juvenile stages.

## Materials and Methods

### Study area

Jeju Island, with a total area of 1,846 km^2^, is a volcanic island standing about 100 km off the south of the Korean Peninsula. The island is elliptical in shape with a major axis 73 km wide and a minor axis 41 km long (Fig. 1). The island is composed mainly of basaltic rocks and a subordinate amount of sedimentary and pyroclastic rocks^27^. The high permeability of the basalt rocks allows the rainfalls to infiltrate readily into subsurface. On the island, most streams are ephemeral and have temporary flows after heavy rain events. Therefore, groundwater is the most important water resource and provides approximately 92% of the water supply for various purposes^39^. However, the groundwater is exposed to contamination sources, especially fertilizers and swine manure, because agricultural land use is very active in the island^40^.

The study area, Jocheon harbor, is located in the northeastern part of Jeju Island (Fig. 1). This area has a mean area of about ~0.32 km^2^ with a mean depth of ~2 m. More than 60 groundwater springs are distributed in Jocheon, and the average discharge rate is 8.8 × 10^4^ m^3^ d^−1^ ^40^.

### Sampling and analyses

Water samples were collected from Jocheon harbor and adjacent groundwater wells in July 2015, August 2015, February 2016, and March 2016 (Fig. 1). Seawater samples from the surface and bottom layers were collected using an electric submersible pump on shipboard. Fresh groundwater samples were collected from groundwater wells along the coastline of Jocheon. Saline groundwater samples were collected from seepage chambers by a scuba diver. The salinities were measured using a portable multi-parameter sensor (Orion star A329, Thermo Scientific) for groundwater and a conductivity–temperature–depth (CTD) profiler (Ocean Seven 304, IDRONAUT Srl) for seawater.

We used a modified Lee-type manual seepage meter to collect the seeping groundwater (saline groundwater)^14^. The seepage chambers were installed in bottom sediments in July 2015 (n = 2), August 2015 (n = 11), and February 2016 (n = 8). The collection bag was attached to a nipple ball valve at the top of the chamber. The collected saline groundwater samples in the empty bag were measured for salinity, ^222^Rn, and nutrients.

In order to analyze the activity of ^222^Rn in groundwater and seawater, about 4 L of water sample was collected in a pre-evacuated glass bottle. After ^222^Rn in water allowed to reach equilibrium with air in a closed air-loop, the activity of ^222^Rn in the air-loop was measured using a radon-in-air monitor (RAD7, Durridge Co. Ltd)^13, 14^. The activity of ^222^Rn in water was calculated using an air-water partitioning factor, which is a function of temperature and salinity^15^.

Groundwater and seawater samples for nutrient analysis were filtered through pre-combusted glass fiber filters (Whatman GF/F, 0.7 µm in pore size, 47 mm in diameter) and stored frozen until analysis. Nutrients, including NH_4_^+^, NO_2_^−^, NO_3_^−^, and PO_4_^3−^, were analyzed using an auto nutrient analyzer (New QuAAtro39, SEAL Analytical). In this study, the sum of NH_4_^+^, NO_2_^−^ and NO_3_^−^ is considered as DIN, and PO_4_^3−^ is considered as DIP.

### Mesocosm experiments

In order to evaluate the effects of SFGD on the growth of green macroalgae *U*. *pertusa*, we conducted *in situ* mesocosm experiments. Thalli of *U. pertusa* for the mesocosem experiments were collected from Jocheon harbor. Then, whole thalli were rinsed gently using 0.22 µm filtered seawater to remove small grazers, epiphytes, and surface contaminants. In order to obtain the sample disks of *U. pertusa*, the adult thalli were cut using a cork-borer (40 mm in diameter) and allowed to recover from respiration by wound for a day. Since fresh *U. pertusa* grows in a diffuse pattern, the samples were cut from random locations over the entire thallus^26^.

Mesocosm experiments were conducted during five days between April 26 and April 30, 2016, in Jocheon harbor. The experimental culture was carried out in a 27 L chamber (30 cm × 30 cm × 30 cm) covered with a polyethylene film. For the experiments, one control seawater (open-ocean seawater) and five different mixtures of the open-ocean seawater and fresh groundwater were prepared. The mixing proportions of fresh groundwater to the open-ocean seawater were 1%, 5%, 10%, 30%, and 50%. Five sample disks of *U. pertusa* were inoculated to each chamber containing 20 L of the seawater mixture. All experiments were incubated under natural temperature and irradiance conditions. The initial temperature, irradiance, salinity, and nutrient concentrations under all experimental conditions were listed in Table 2. The diameter of the sample disks was measured daily using a vernier caliper. The growth rate (µ; d^−1^) of *U. pertusa* was calculated using Eq. 2^26^:

2 $$\mu =(1/{\rm{t}})\,\cdot \,\mathrm{ln}({{\rm{A}}}_{{\rm{t}}}/{{\rm{A}}}_{{\rm{o}}})$$

where t is the incubation time in days, A_o_ is the initial area of thallus, and A_t_ is the area after t days.

## Electronic supplementary material

Supplementary Information
