# Supplementary material for: Green tide development associated with submarine groundwater discharge in a coastal harbor, Jeju, Korea
Source: Sci Rep. 2017 Jul 24;7:6325. doi: 10.1038/s41598-017-06711-0 (PMC5524685; doi:10.1038/s41598-017-06711-0)
Supplement: Supplementary file 1 — Supplementary Information [file 41598_2017_6711_MOESM1_ESM.pdf]

Supplementary Information

Green tide development associated with submarine groundwater discharge in a coastal harbor, Jeju, Korea

Hyeong Kyu Kwon[1], Hyekyung Kang[1], Yong Hwa Oh[1], Sang Rul Park[2] & Guebuem Kim[1*]

[1]School of Earth and Environmental Sciences, Seoul National University, Seoul 08826, Republic of Korea

[2]Department of Marine Life Sciences, Jeju National University, Jeju 63243, Republic of Korea

*Corresponding author: gkim@snu.ac.kr

Table S1. The concentrations of salinity, ^{222}Rn, and nutrients in fresh groundwater, saline groundwater, harbor seawater, and open-ocean seawater in July 2015 in Jocheon, Jeju Island (S = surface, B = bottom and n.d. = non detect). Uncertainties of ^{222}Rn activities are based on 1σ counting error propagations.

Sample		Salinity	^{222}Rn (Bq m^{-3})	Nutrients (μM)		N:P ratio
				DIN	DIP	
Fresh groundwater	F7	2.2	5920±60	400	2.2	182
	F8	4.6	-	390	2.3	170
	F9	0.3	4920±70	460	2.3	200
Saline groundwater	S2	31.5	-	12	0.32	37
	S6	31.5	-	7.7	0.35	22
	S9	24.8	-	99	0.80	124
	S10	25.4	-	83	0.71	117
Harbor seawater	H2 (S)	29.8	250±10	43	0.27	158
	H2 (B)	32.1	98±7	10	0.02	505
	H3 (S)	27.8	740±20	70	0.38	184
	H3 (B)	32.0	89±7	13	0.07	180
	H9 (S)	23.8	590±30	112	0.22	509
	H16 (S)	30.5	230±20	39	0.22	175
	H17 (S)	29.8	100±11	73	0.43	170
	H17 (B)	32.7	71±7	4.8	0.02	240
	H19 (S)	25.8	270±10	81	0.08	1013
	H21 (S)	32.3	44±9	4.5	n.d.	-
	H21 (B)	32.7	20±3	4.5	0.09	50
	H22 (S)	31.7	-	11	n.d.	-
	H23 (S)	28.4	370±20	67	0.39	172
	H23 (B)	32.4	12±3	4.2	n.d.	-
	H24 (S)	31.7	200±10	40	0.23	173
	H24 (B)	32.3	23±7	4.8	n.d.	-
	H27 (S)	29.6	140±10	55	0.35	157
	H27 (B)	32.4	110±10	6.1	0.09	68
	H28 (S)	29.9	180±10	72	0.44	164
	H28 (B)	32.4	43±4	6.2	0.05	124
Open-ocean seawater	O1 (S)	32.4	23±4	n.d.	n.d.	-
	O1 (B)	32.6	16±6	0.88	n.d.	-

Table S2. The concentrations of salinity, ^{222}Rn, and nutrients in fresh groundwater, saline groundwater, harbor seawater, and open-ocean seawater in August 2015 in Jocheon, Jeju Island (S = surface, B = bottom and n.d. = not detected). Uncertainties of ^{222}Rn activities are based on 1σ counting error propagations.

Sample		Salinity	^{222}Rn ($Bq\ m^{-3}$)	Nutrients (μM)		N:P ratio
				DIN	DIP	
Fresh groundwater	F1	1.6	2490±40	490	2.4	204
	F2	0.2	1550±30	580	2.8	207
	F3	0.2	2060±40	490	4.3	114
	F4	2.6	3510±60	380	2.4	158
	F9	0.2	8270±190	510	2.2	232
	F10	0.4	-	650	2.5	260
Saline groundwater	S2	15.0	1070±20	225	3.5	64
	S3	31.6	85±17	32	1.7	19
	S4	32.5	69±5	5.1	0.43	12
	S5	32.7	84±15	9.0	0.48	19
	S6	33.0	130±10	4.4	0.33	13
	S7	32.7	30±58	12	0.60	19
	S11	27.8	740±40	56	n.d.	-
	S12	30.6	130±10	18	0.07	250
	S13	31.4	150±20	16	0.06	262
	S14	30.7	54±5	15	0.11	132
Harbor seawater	H1 (S)	31.7	60±5	9.8	n.d.	-
	H2 (S)	31.9	60±5	7.9	n.d.	-
	H5 (S)	28.6	81±5	42	0.08	521
	H6 (S)	28.4	240±10	49	0.08	618
	H7 (S)	27.9	200±10	88	0.26	338
	H8 (S)	30.5	140±10	55	0.07	786
	H10 (S)	25.2	120±10	41	n.d.	-
	H11 (S)	28.4	160±10	23	0.04	575
	H12 (S)	29.2	130±10	13	n.d.	-
	H13 (S)	31.3	100±10	18	n.d.	-
	H14 (S)	28.7	290±10	55	0.09	611
	H15 (S)	28.4	190±10	51	0.09	567
	H16 (S)	29.0	210±10	37	0.05	738
	H18 (S)	31.9	26±7	17	n.d.	-
	H20 (S)	32.1	25±10	6.5	n.d.	-
	H21 (S)	32.0	16±3	2.5	n.d.	-
	H22 (S)	32.1	10±3	2.9	n.d.	-
	H23 (S)	32.1	13±5	2.4	n.d.	-
	H24 (S)	32.2	26±6	2.2	n.d.	-
	H25 (S)	31.6	40±5	7.7	n.d.	-
	H26 (S)	32.3	24±3	0.9	n.d.	-
	H28 (S)	30.3	68±5	15	n.d.	-
Open-ocean seawater	O1 (S)	32.5	4±2	0.8	n.d.	-

Table S3. The concentrations of salinity, ^{222}Rn, and nutrients in fresh groundwater, saline groundwater, harbor seawater, and open-ocean seawater in February 2016 in Jocheon, Jeju Island (S = surface, B = bottom and n.d. = not detected). Uncertainties of ^{222}Rn activities are based on 1σ counting error propagations.

**Sample**		**Salinity**	**^{222}Rn (Bq m^{-3})**	**Nutrients (μM)**		**N:P ratio**
				**DIN**	**DIP**	
Fresh groundwater	F1	0.8	1700±30	400	2.9	138
	F2	0.7	1400±40	430	3.0	143
	F3	0.3	1910±30	440	4.0	110
	F4	0.9	5720±90	400	2.3	182
	F5	1.0	6310±80	480	2.0	240
	F6	0.2	6100±70	440	2.3	191
	F7	1.0	5690±60	460	2.0	230
	F8	0.4	3860±40	490	2.2	223
	F9	1.4	3540±40	490	1.9	258
Saline groundwater	S1	34.1	-	9.2	0.47	20
	S2	34.0	-	13	0.73	18
	S3	33.8	-	11	0.53	21
	S8	33.8	-	11	0.36	32
	S11	34.1	-	10	0.46	22
	S13	34.4	-	6.5	0.48	13
	S14	34.2	-	10	0.48	21
Harbor seawater	H2 (S)	32.9	100±10	19	0.34	54
	H2 (B)	34.1	13±3	6.9	0.36	19
	H3 (S)	33.2	6±4	8.1	0.37	22
	H3 (B)	34.4	13±5	7.5	0.37	20
	H5 (S)	31.8	120±10	32	0.36	89
	H5 (B)	34.0	100±10	27	0.40	68
	H6 (S)	31.4	160±10	44	0.34	129
	H6 (B)	31.7	140±10	27	0.41	67
	H8 (S)	31.0	260±10	41	0.34	121
	H8 (B)	32.9	106±9	23	0.41	57
	H10 (S)	33.4	28±4	11	0.34	31
	H10 (B)	34.4	31±5	6.7	0.30	22
	H14 (S)	34.1	40±5	5.9	0.34	17
	H14 (B)	34.3	44±6	11	0.40	28
	H16 (S)	34.0	110±10	25	0.36	70
	H16 (B)	34.4	5±3	7.1	0.36	20
	H21 (S)	34.0	20±5	7.3	0.39	19
	H21 (B)	34.5	8±4	6.4	0.34	19
	H24 (S)	34.3	6±3	8.6	0.38	23
	H24 (B)	34.5	12±4	7.2	0.35	21
Open-ocean seawater	O2 (S)	34.6	4±3	6.0	0.39	15
	O3 (S)	34.6	21±4	5.1	0.38	13
	O4 (S)	34.5	24±5	5.1	0.41	12

Table S4. The concentrations of salinity, ^{222}Rn, and nutrients in fresh groundwater, harbor seawater, and open-ocean seawater in March 2016 in Jocheon, Jeju Island (S = surface, B = bottom and n.d. = not detected). Uncertainties of ^{222}Rn activities are based on 1σ counting error propagations.

Sample		Salinity	^{222}Rn (Bq m^{-3})	Nutrients (μM)		N:P ratio
				DIN	DIP	
Fresh groundwater	F1	0.2	2780±50	410	2.6	158
	F2	0.2	2170±40	430	2.6	165
	F3	0.2	1880±40	410	3.8	108
	F4	0.9	4840±80	390	2.8	139
	F7	0.2	4260±60	460	2.5	184
	F8	0.2	3360±50	480	2.3	209
	F9	0.2	5360±100	480	2.4	200
Harbor seawater	H2 (S)	33.2	160±10	20	0.06	337
	H2 (B)	34.6	47±8	0.8	n.d.	-
	H3 (S)	32.8	160±20	14	0.10	142
	H3 (B)	34.2	33±5	1.7	0.02	85
	H4 (S)	29.4	430±20	35	0.14	251
	H4 (B)	34.4	55±7	1.3	0.03	43
	H6 (S)	27.9	560±20	63	0.26	242
	H8 (S)	30.7	260±10	45	0.12	378
	H10 (S)	31.7	240±10	35	0.14	252
	H10 (B)	34.6	29±10	0.5	0.01	50
	H14 (S)	31.3	-	50	0.14	357
	H14 (B)	34.6	-	0.5	0.07	7
	H16 (S)	31.2	140±10	18	0.07	254
	H16 (B)	34.6	23±6	1.1	0.02	55
	H21 (S)	33.7	51±12	11	0.03	373
	H21 (B)	34.7	17±4	0.3	n.d.	-
	H24 (S)	34.2	58±7	4.6	0.01	460
	H24 (B)	34.6	19±9	0.5	0.01	50
Open-ocean seawater	O2 (S)	34.8	4±4	1.0	0.02	50
	O3 (S)	34.7	19±4	0.6	0.01	60
	O4 (S)	34.7	14±7	0.7	n.d.	-

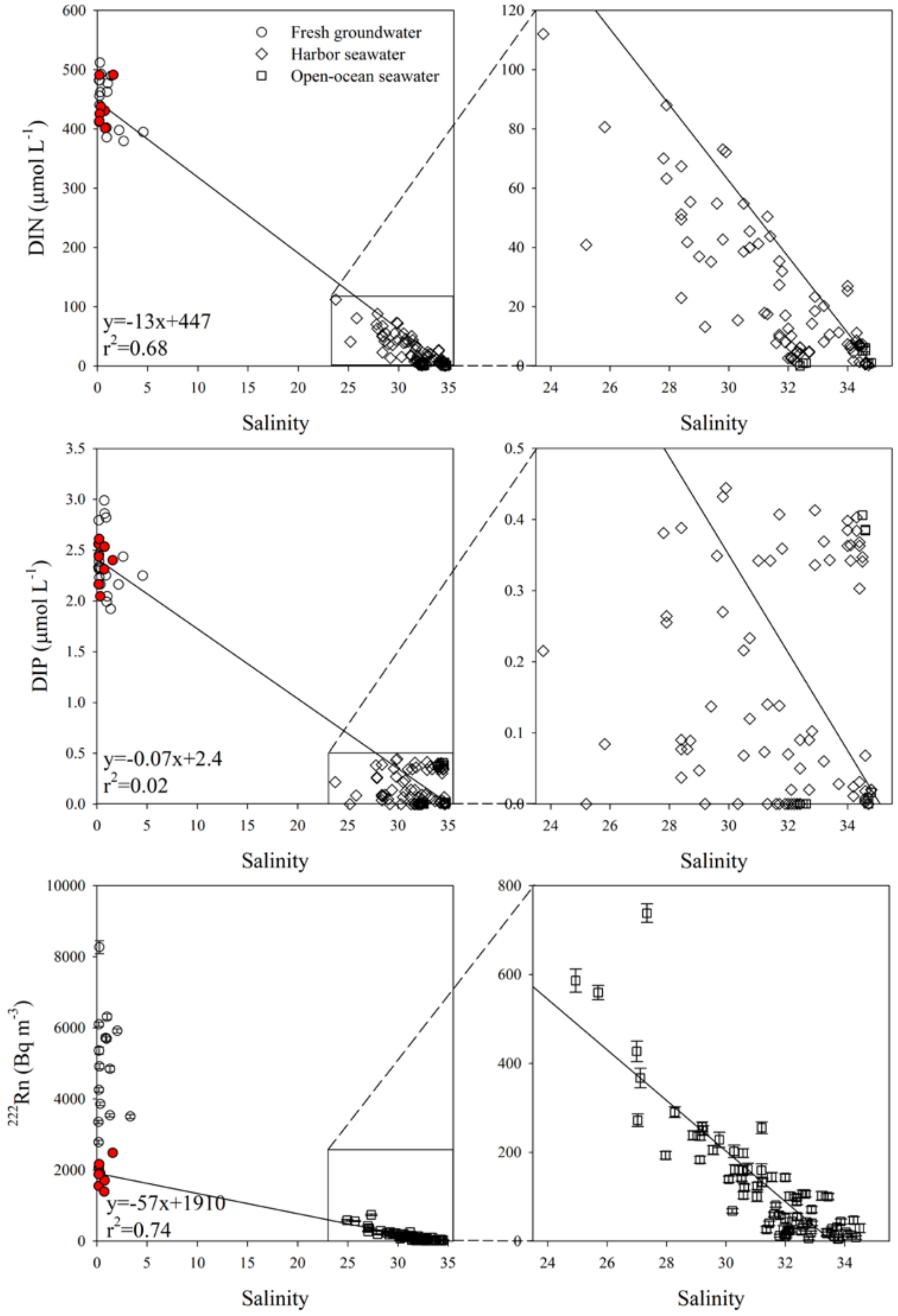

Figure S1. Plots of salinity versus DIN, DIP, and ^{222}Rn concentrations in fresh groundwater and seawater. The solid line indicates the conservative mixing between the two end-members (fresh groundwater and seawater). The red circles represent the major springs based on the ^{222}Rn extrapolation from harbor seawater.

References

(1) Ullman, W. J. & Sandstrom, M. W. Dissolved nutrient fluxes from the nearshore sediments of Bowling Green Bay, central Great Barrier Reef lagoon (Australia). *Estuar. Coast. Shelf Sci.* **24**, 289-303 (1987).

(2) Lerat, Y., Lasserre, P. & Corre, P. Seasonal changes in pore water concentrations of nutrients and their diffusive fluxes at the sediment-water interface. *J. Exp. Mar. Biol. Ecol.* **135**, 135-160 (1990).

(3) Kuwae, T., Hosokawa, Y. & Eguchi, N. Dissolved inorganic nitrogen cycling in Banzu intertidal sand-flat, Japan. *Mangr. Salt Marsh.* **2**, 167-175 (1998).

(4) Knoppers, B. *et al.* In situ measurements of benthic primary production, respiration and nutrient fluxes in a hypersaline coastal lagoon of SE Brazil. *Rev. Bra. Oceanogr.* **44**, 155–165 (1996).

(5) Stimson, J. & Larned, S. T. Nitrogen efflux from the sediments of a subtropical bay and the potential contribution to macroalgal nutrient requirements. *J. Exp. Mar. Biol. Ecol.* **252**, 159–180 (2000).

(6) Rasheed, M., Badran, M. I. & Huettel, M. Influence of sediment permeability and mineral composition on organic matter degradation in three sediments from the Gulf of Aqaba, Red Sea. *Estuar. Coast. Shelf Sci.* **57**, 369-384 (2003).

(7) Dixon, L. K., Murphy, P. J., Becker, N. M. & Charniga, C. M. The potential role of benthic nutrient flux in support of *Karenia* blooms in west Florida (USA) estuaries and the nearshore Gulf of Mexico. *Harmful Algae* **38**, 30-39 (2014).

(8) Sundback, K. *et al.* Importance of benthic nutrient regeneration during initiation of macroalgal blooms in shallow bays. *Mar. Ecol. Prog. Ser.* **246**, 115-126 (2003).

(9) Ospina-Alvarez, N. *et al.* Exchange of nutrients across the sediment-water interface in intertidal rea systems (SW Europe). *J. Sea Res.* **85**, 349-358 (2014).
